# Potential of [^11^C](*R*)-PK11195 PET Imaging for Evaluating Tumor Inflammation: A Murine Mammary Tumor Model

**DOI:** 10.3390/pharmaceutics14122715

**Published:** 2022-12-04

**Authors:** Aline Morais de Souza, Caroline Cristiano Real, Mara de Souza Junqueira, Larissa Estessi de Souza, Fábio Luiz Navarro Marques, Carlos Alberto Buchpiguel, Roger Chammas, Marcelo Tatit Sapienza, Daniele de Paula Faria

**Affiliations:** 1 Laboratory of Nuclear Medicine (LIM 43), Department of Radiology and Oncology, Faculdade de Medicina FMUSP, Universidade de Sao Paulo, Sao Paulo 05403-911, Brazil; 2 Department of Nuclear Medicine and PET Center, Aarhus University Hospital, DK-8200 Aarhus, Denmark; 3 Centro de Investigação Translacional em Oncologia (CTO), Instituto do Câncer de Sao Paulo (ICESP), Hospital das Clínicas HCFMUSP, Faculdade de Medicina, Universidade de Sao Paulo, Sao Paulo 01246-000, Brazil

**Keywords:** [^11^C](*R*)-PK11195, TSPO, 4T1 cells, macrophages, positron emission tomography, breast cancer, inflammation

## Abstract

Background: Breast tumor inflammation is an immunological process that occurs mainly by mediation of Tumor-Associated Macrophages (TAM). Aiming for a specific measurement of tumor inflammation, the current study evaluated the potential of Positron Emission Tomography (PET) imaging with [^11^C](*R*)-PK11195 to evaluate tumor inflammation in a mammary tumor animal model. Methods: Female Balb/C mice were inoculated with 4T1 cells. The PET imaging with [^11^C](*R*)-PK11195 and [^18^F]FDG was acquired 3 days, 1 week, and 2 weeks after cell inoculation. Results: The [^11^C](*R*)-PK11195 tumor uptake increased from 3 days to 1 week, and decreased at 2 weeks after cell inoculation, as opposed to the [^18^F]FDG uptake, which showed a slight decrease in uptake at 1 week and increased uptake at 2 weeks. In the control group, no significant differences occurred in tracer uptake over time. Tumor uptake of both radiopharmaceuticals is more expressed in tumor edge regions, with greater intensity at 2 weeks, as demonstrated by [^11^C](*R*)-PK11195 autoradiography and immunofluorescence with TSPO antibodies and CD86 pro-inflammatory phenotype. Conclusion: The [^11^C](*R*)-PK11195 was able to identify heterogeneous tumor inflammation in a murine model of breast cancer and the uptake varied according to tumor size. Together with the glycolytic marker [^18^F]FDG, molecular imaging with [^11^C](*R*)-PK11195 may provide a better characterization of inflammatory responses in cancer.

## 1. Introduction

The tumor microenvironment (TME) contains immune cells, such as mast cells, dendritic cells (DC), natural killers (NK), and macrophages. Macrophages are the main initiators and maintainers of the chronic inflammation present in the TME, through production and secretion of inflammatory mediators [1]. The presence of chronic inflammation, characterized by tissue remodeling, neoangiogenesis, and macrophage infiltration is favorable to tumor onset and progression [2,3,4].

In vivo imaging techniques, such as Positron Emission Tomography (PET), would be a helpful technique for evaluating Tumor-Associated Macrophages (TAM) in the TME with the use of specific biomarkers, opening possibilities for the evaluation and follow-up of tumors. The [^18^F]FDG PET imaging measures glycolytic metabolism, and is widely used in cancer staging and metastasis detection studies. The [^18^F]FDG uptake occurs not only in tumor cells but also in high-metabolic cells, such as activated macrophages [5].

The molecule (*R*)-1-(2-chlorophenyl)-N-methyl-N-1-(1-methyl-propyl]-3-isoquinoline carboxamide, namely (*R*)-PK11195, is a synthetic substance, initially implemented to evaluate inflammation in different organs due to its affinity for translocator protein 18 kDa (TSPO) [6]. The TSPO is located in the mitochondrial membrane [7] and is overexpressed [8] in activated glial cells in neuroinflammation models [9,10,11,12,13,14,15] and in different tumor cell lines [16], participating in the modulation of cell proliferation and tumorigenesis [17]. Positron Emission Tomography imaging using [^11^C](*R*)-PK11195 has been widely used in molecular imaging to evaluate neuroinflammation [18,19,20], but its potential to evaluate tumor inflammation needs to be investigated. Previous studies used this tracer to evaluate inflammation in brain tumors [21,22,23], and recent studies proposed the investigation of TSPO expression as a non-invasive tool to understand and evaluate the mechanisms involved in the TME [24].

The present study proposes to evaluate the expression of TSPO and correlate it with cell metabolism in the TME of murine mammary tumors, using [^11^C](*R*)-PK11195 and [^18^F]FDG PET imaging, respectively. Our hypothesis is that there is overexpression of TSPO due to tumor development and inflammatory infiltrate, not necessarily correlated with increased uptake of [^18^F]FDG.

## 2. Materials and Methods

### 2.1. Ethical Approval

All animal procedures were in accordance with the guidelines of the National Council for Animal Experimentation Control and were approved by the Ethical Committee for Animal Use of the University of Sao Paulo Medical School (protocol number: 992/2018).

### 2.2. [^11^C](R)-PK11195 Radiosynthesis

The [^11^C](*R*)-PK11195 was produced in the Center of Nuclear Medicine of the Institute of Radiology of Hospital das Clinicas of the University of Sao Paulo Medical School (HCFMUSP). Briefly, [^11^C](*R*)-PK11195 was synthesized by trapping [^11^C]methyl iodide in a solution of 1 mg (*R*)-N-desmethyl-PK11195 and 30 mg of potassium hydroxide in 300 μL of dry dimethyl sulfoxide. The reaction mixture was allowed to react for 1 min at 40 °C. The mixture was neutralized with 1 M HCl and filtered through a 1.2 μm Millex HV filter. The filtrate was purified by High Performance Liquid Chromatography (HPLC) using a Luna C18 column (250 × 10 mm, 5 μm) (Phenomenex^®^, Torrance, CA, USA) with water:ethanol (40:60) as mobile phase (flow rate 5 mL/ min). The fraction was collected at the retention time of 10 ± 1 min before being diluted in 50 mL of water and passed through a SepPak^®^ C18 light cartridge (Waters, Barueri, Brazil). The cartridge was eluted with 1 mL of ethanol and washed with 10 mL of saline.

Quality control was performed by HPLC using a Nova-Pak^®^ C18 column (150 × 3.9 mm, 5 µm) (Waters, Brazil) with water:acetonitrile (55:45) mobile phase (flow rate 1 mL/min). The final product was approved in all quality control tests (appearance, pH, chemical and radiochemical purity, residual solvents, radionuclide identity, specific activity, endotoxins, and sterility).

### 2.3. Animals

Specific pathogen-free seven-week-old female Balb/C mice (*n* = 27) were obtained from the animal facility of the University of Sao Paulo Medical School. The mice were maintained at a controlled temperature of 22 ± 2 °C in a 12 h light/dark cycle, with humidity of 55% ± 5%, and food and water provided ad libitum.

The animals received a standard diet (Nuvilab CR-1 irradiated, Quintia S.A.), containing Ground Whole Corn, Soybean Bran, Wheat Bran, Calcium Carbonate, Dicalcium Phosphate, Sodium Chloride (Common Salt), Vitamin A, Vitamin D3, Vitamin E, Vitamin K3, Vitamin B1, Vitamin B2, Vitamin B6, Vitamin B12, niacin, calcium pantothenate, folic acid, biotin, choline chloride, iron sulfate, manganese monoxide, zinc oxide, copper sulfate, calcium iodate, sodium selenite, cobalt sulfate, lysine, methionine, and BHT, more detailed information is provided in the Supplementary Material.

The animals were acclimated for at least 1 week before starting the experiments. Animals with a tumor were carefully monitored for signs of distress and discomfort and were humanely euthanized when these were confirmed.

As breast tumors are more recurrent in women, the tumor model was induced in female animals for this experiment, and the choice of using 4T1 lineage cells was based on availability and TSPO expression.

### 2.4. Cell Culture

A cryotube containing 1 mL of 4T1 murine tumor cells, kept frozen at −80 °C, was quickly thawed with the aid of a water bath (37 °C). Cells were transferred to a culture bottle in a laminar flow hood, with the addition of RPMI 1640 medium (Roswell Park Memorial Institute), supplemented with 10% fetal bovine serum inactivated by endotoxin-free heat in PBS buffer solution, previously heated in a water bath (37 °C). The bottle containing the cells was kept in a humidified incubator at 37 °C and 5% CO_2_. Cell growth and viability were monitored daily, with transfer to other bottles until reaching the necessary amount for inoculation: aliquots (1 × 10^5^ cells / 100 μL) x number of animals to be injected + 3 extra aliquots. Cell counting and viability were assessed using the Trypan Blue exclusion test in a Neubauer camera 9020-01 (HBG, Giessen, Germany) and an Eclipse TS100 microscope (Nikon, Tokyo, Japan).

### 2.5. Animal Model Induction and Groups

The 27 mice in this study were divided into two groups: (1) animals inoculated with 4T1 tumor cells (*n* = 23), herein called tumor group and, (2) animals that received an injection of RPMI culture medium, herein called control group (*n* = 10), where four of the animals received only an injection of medium in the left breast and the other six animals received medium in the left breast and tumor cells in the right breast, therefore being part of both groups.

The animals from the tumor group were anesthetized with 2–3% isoflurane in oxygen and were inoculated subcutaneously with 1 × 10^4^ 4T1 cells in RPMI 1640 serum-free medium into the upper right breast. Tumors were measured every 2 days with a caliper and the tumor volume was calculated using the following formula: V = 0.52 × d2 × D, where d2 is the shortest diameter and D is the longest diameter. From the tumor cells inoculated animals, all presented tumor growth and were included in the study (there was no animal exclusion because of no tumor growing in the tumor group).

Animals from the control group were injected into the upper left breast with a volume of 100 μL of RPMI 1640 medium, to evaluate whether the mechanical injury caused by the syringe used for cell injection could have any influence on the tracer uptake at the evaluated time points.

**Figure 1:**
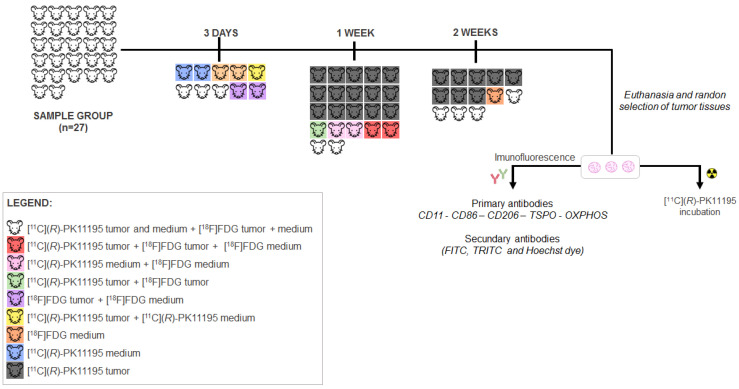
Experimental study design and the divisions/sub-groups at each time point, illustrated by different colors. Note: As the main scientific question was related to the [^11^C](*R*)-PK11195 uptake in the tumor, the sample size for this tracer was bigger than for [^18^F]FDG, as higher variations in the results were expected due to tumor growing heterogeneity.

Figure 1 shows the study design and how the animals were divided for PET acquisitions with [^11^C](*R*)-PK11195 and [^18^F]FDG and histological analysis.

### 2.6. PET Imaging

The [^11^C](*R*)-PK11195 and/or [^18^F]FDG PET images were acquired at three time points after tumor cell inoculation and/or medium injection: 3 days, 1 week, and 2 weeks.

The mice (*n* = 20) were anesthetized with 2–3% isoflurane, administered in the caudal vein with 50.77 ± 6.09 MBq of [^11^C](*R*)-PK11195, before being left in the cage for 30 min, prior to imaging acquisition. Similar protocols were used for [^18^F]FDG, in a group of six mice, which were injected with 37.38 ± 1.51 MBq and the animals were left in the cage for 45 min. For imaging acquisition, animals were anesthetized again and were positioned with the tumor or inoculation site in the center of the field of view (FOV) of the PET/CT imaging equipment for small animals (Triumph^®^ II Trimodality System, CA, USA). For both tracers, image acquisition was performed for 30 min.

After PET acquisition, computed tomography (CT) images were acquired to be used as an anatomical reference of the tumor for PET analysis, with 512 projections, 45 kVp, 400 μA, and 1.3× magnification.

**Figure 2:**
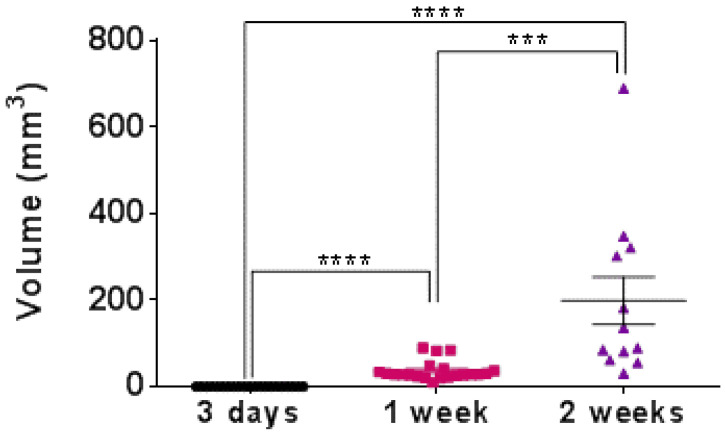
Tumor volumes were measured at 3 days, 1 week, and 2 weeks after 4T1 cell inoculation (**** *p* < 0.0001, *** *p* = 0.0005, unpaired Student *t*-test). Note: The volume was considered zero at the 3-day time point due to the undetectable volume at the cell injection site.

The PET images were reconstructed by the 3D OSEM algorithm (20 iterations and 4 subsets) and merged with the CT images in PMOD™ software. Fusion, in addition to providing anatomical information, helped to manually draw the volume of interest (VOI) on the tumors in the different anatomical planes (axial, coronal, or sagittal) (Figure 2). An extra VOI was drawn in the muscle region. The PET images were quantified by the mean Standardized Uptake Value (SUVmean) within the VOIs, and also by the tumor/muscle ratio (TMR) of the SUVmean; SUV: [tissue activity concentration (kBq/mL) (mean) × body weight(g)]/[injected dose (kBq)] for each region. A tissue density of 1 g/mL was assumed.

### 2.7. Ex-Vivo Autoradiography

The autoradiography technique was performed using randomly collected tissues. The procedure was performed for comparison with PET image data. After PET imaging (60 min after [^11^C](*R*)-PK11195 injection), the mice were deeply anesthetized (5% isoflurane in 100% oxygen) and euthanized by heart excision, and the tumors were extracted.

The tumors were frozen at the Optimal Cutting Temperature (O.C.T.) directly on the metallic base of the cryostat, before being cut in 40 μm sections and collected on histological slides. At room temperature, the slides were quickly dried with nitrogen (N_2_). The dried slides were exposed on a phosphorus plate (Fujifilm Corporation, Tokyo, Japan) for two hours in a dark place, away from any light source. After the exposure time, still in the dark, the phosphorus plate was placed on the photoluminescence scanner (Typhoon FLA 9500—GE Healthcare Life Sciences, Uppsala, Sweden).

### 2.8. Immunofluorescence

Immunofluorescence was performed using randomly collected tissues. The procedure was performed for comparison with PET image data. The mice were deeply anesthetized with 5% isoflurane in 100% oxygen and euthanized by extirpation of the heart. The tumors were dissected and included in a mold with melted paraffin (65 to 70 °C), cut (4–5 μm tissues) and collected on previously gelatinized slides. Slides were deparaffinized and rehydrated with xylol, 100% ethanol, 95% ethanol, 70% ethanol, and distilled water, 2 times (consecutively 5 min each).

For antigen recovery, slides were left in 10 mM citrate buffer pH 6.0 (90–100 °C/10 min) and then washed with water at room temperature/10 min. Subsequently, they were incubated with 0.1 M phosphate buffer containing 0.25% triton-x (room temperature/10 min), washed with 0.1 M phosphate buffer (3 times, 5 min each), and incubated with 0.15 M glycine in 0.1 M phosphate buffer (30 min, room temperature) and washed with 0.1 M phosphate buffer (3 times, 5 min).

To block non-specific binding, slides were incubated in 5% albumin (bovine) in 0.1 M phosphate buffer + 10% normal goat serum (005-000-121—the Jackson Laboratory, USA) in 0.1 M phosphate buffer (30 min, room temperature).

Immunofluorescence was performed with the primary antibodies diluted in 0.1 M phosphate buffer and prepared for single or double staining, as follows: 1:250 mice anti-rabbit OXPHOS (a mixture of 5 monoclonal antibodies that recognize proteins associated with the phosphorylation process) (ab110413—Abcam, Waltham, MA, USA); or (a) 1:50 CD11 integrin alpha-m/beta-2 marker (Jackson Laboratory, Bar Harbor, ME, USA) + 1:100 rabbit anti-human TSPO marker (LS-B14234—LSBio, Seattle, WA, USA); or (b) 1:250 mouse anti-human for CD86 (macrophage marker M1) (ab213044—Abcam, USA) + 1:250 rabbit anti-mouse for CD206 or anti-mannose receptor (macrophage marker M2) (ab64693—Abcam, USA).

Each slide was incubated with 200μL of primary antibody solution in a humidified chamber in the dark (room temperature, 1–2 hours) and then for 12 hours at 4 °C. Subsequently, the slides were washed three times with 0.1 M phosphate buffer (5 min each) and incubated with 200 μL of secondary antibody solution: (1) single stain: goat anti-rabbit IgG conjugated to FITC (ab6717-1—Abcam, USA); (1: 100); and (2) dual staining: 1:100 rhodamine fluorophore-conjugated goat anti-mouse (TRITC) IgG (T-7782—Sigma Aldrich, USA) + 1:100 fluorescein fluorophore-conjugated goat anti-rabbit IgG (FITC) (ab6717-1—Abcam, USA);

The slides were incubated in a humid and dark chamber for 1 h (room temperature) and then washed with 0.1 M phosphate buffer (3 times, 5 min each). Slides were then incubated for 30 min with Hoescht DNA/nuclear marker (33258 H3569—Invitrogen, Waltham, MA, USA) diluted in 0.1 M phosphate buffer (1:500). Finally, the slides were washed with 0.1 M phosphate buffer (3 times, 5 min each).

The slides were covered with a 1:1 glycerol solution in 0.1 M phosphate buffer and a coverslip to preserve the slices. The slides were evaluated under a microscope (Eclipse E100—Nikon, Japan) coupled to the capture camera (DXM1200—Nikon, Japan) for digital images of the histological sections and, were then stored in the dark at 4 °C.

### 2.9. Protein Extraction and Western Blot

Western Blot was performed to confirm the expression of TSPO in 4T1 cells and to compare with two other lineages of breast cancer (MDA-MB231 and MCF7). Cells were trypsinized and centrifuged at 2000 rpm for 2 min, and proteins were extracted and submitted to the procedure as described by Tortelli et al. [25]. The samples were visualized with the chemiluminescent substrate ECL (GE Healthcare) followed by imaging with Image Quant (GE Healthcare).

### 2.10. Statistical Analysis

Statistical analyses were performed using Graphpad Prism (GraphPad Software, v.8, San Diego, CA, USA). Data are described as means ± standard error. Differences were considered statistically significant when *p* < 0.05. Tumoral uptake data (SUVmean) of [^11^C](*R*)-PK11195 and [^18^F]FDG at 3 days, 1 week, and 2 weeks after 4T1 cells inoculation were statistically analyzed using the non-parametric Mann–Whitney U Test (non-paired data) and tumor growth using the parametric unpaired Student’s *t*-test. The decision to use parametric or non-parametric tests was made based on data normality, which was defined by the Kolmogorov–Smirnov test.

## 3. Results

### 3.1. Tumor Growth

The tumor was not measurable in the first three days after inoculation due to the undetectable volume (*n* = 23) and was, therefore, considered as zero volume. At 1 week, the tumor (*n* = 21) volume was 36.4 ± 4.85 mm^3^ and at 2 weeks (*n* = 12) 198.6 ± 55.0 mm^3^ (>500% increase), as presented in Figure 2.

### 3.2. [^11^C](R)-PK11195 and [^18^F]FDG Uptake

**Figure 3:**
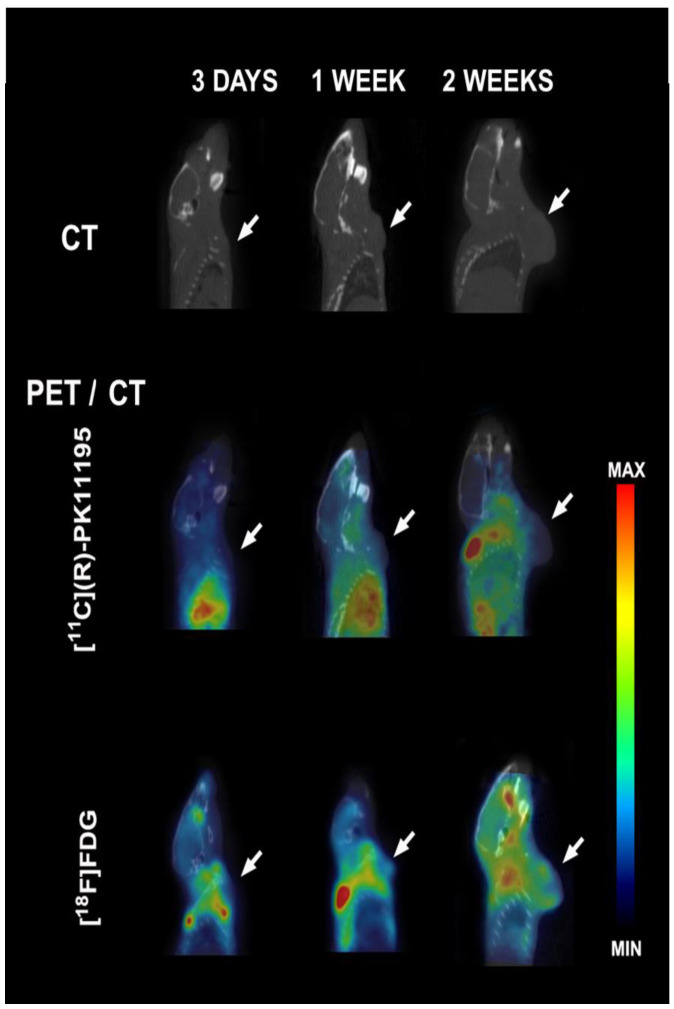
Illustrative [^11^C](*R*)-PK11195 and [^18^F]FDG PET images fused with anatomic reference (CT image). The white arrows indicate the tumor site.

The [^11^C](*R*)-PK11195 and [^18^F]FDG PET images were acquired at 3 days, 1 week, and 2 weeks after 4T1 cell inoculation. Figure 3 illustrates the [^11^C](*R*)-PK11195 PET, [^18^F]FDG PET, and CT images of the same mouse.

**Figure 4:**
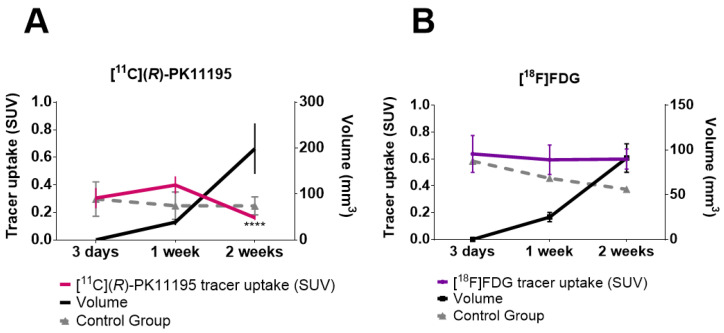
Tumor volume and tracer uptake: (**A**) [^11^C](*R*)-PK11195 uptake in tumor group (red) at 3 days (*n* = 4), 1 week (*n* = 20), and 2 weeks (*n* = 12), and in the control group (gray) at 3 days (*n* = 6), 1 week (*n* = 4), and 2 weeks (*n* = 4); and (**B**) [^18^F]FDG PET uptake in the tumor group (purple) at 3 days (*n* = 5), 1 week (*n* = 5), and 2 weeks (*n* = 4), and in the control group (gray) at 3 days (*n* = 7), 1 week (*n* = 6), and 2 weeks (*n* = 6). (**** *p* < 0.0001; decreased [^11^C](*R*)-PK11195 uptake in 2 weeks compared to 1 week time point, Mann–Whitney U test).

Figure 4 represents the tumor uptake of [^11^C](*R*)-PK11195 (A) and [^18^F]FDG (B) at 3 days, 1 week, and 2 weeks after 4T1 cell inoculation.

The [^11^C](*R*)-PK11195 uptake in the tumor group was 0.30 ± 0.07 at 3 days, increased to 0.40 ± 0.06 at 1 week (*p* = 0.8), and decreased to 0.17 ± 0.02 at 2 weeks after cell inoculation (*p* < 0.0001 compared to 1 week). The [^18^F]FDG uptake revealed no difference between 3 days, 1 week (*p* = 0.94), and 2 weeks (*p* = 0.71), with SUV values of 0.64 ± 0.14, 0.59 ± 0.11, and 0.60 ± 0.07, respectively.

In the control group, the [^11^C](R)-PK11195 uptake was 0.29 ± 0.06 at 3 days, 0.25 ± 0.05 at 1 week and 0.25 ± 0.03 at 2 weeks, and for [^18^F]FDG was 0.59 ± 0.03 at 3 days, 0.46 ± 0.06 at 1 week, and 0.37 ± 0.04 at 2 weeks after inoculation. Comparing the control group and the tumor group, there was no significant difference for either tracer.

**Figure 5:**
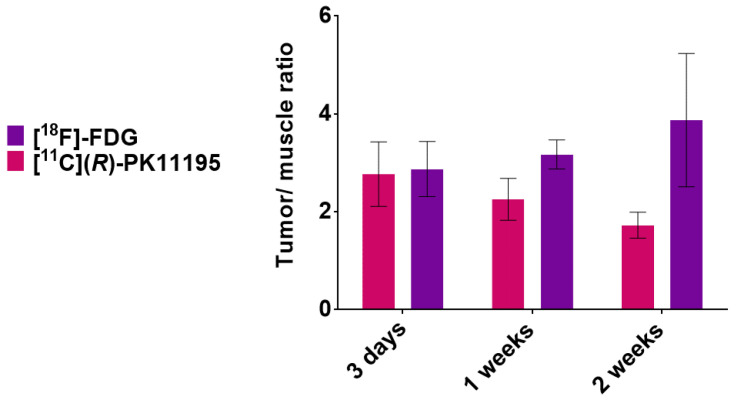
Tumor/muscle uptake ratio: the [^11^C](*R*)-PK11195 tumor/muscle uptake ratio at 3 days (*n* = 4), 1 week (*n* = 20), and 2 weeks (*n* = 12), and the [^18^F]FDG tumor/muscle uptake ratio at 3 days (*n* = 5), 1 week (*n* = 5), and 2 weeks (*n* = 4), (Mann–Whitney U test).

Figure 5 shows the [^11^C](*R*)-PK11195 and [^18^F]FDG tumor/muscle ratios (TMR). The [^11^C](*R*)-PK11195 TMR was 2.8 ± 0.66 at 3 days, 2.3 ± 0.43 at 1 week (*p* = 0.38), and 1.7 ± 0.26 at 2 weeks (*p* = 0.47) and the [^18^F]FDG TMR was 2.9 ± 0.56 at 3 days, 3.2 ± 0.30 at 1 week (*p* = 0.53), and 3.9 ± 1.36 at 2 weeks after inoculation (*p* > 0.9999).

### 3.3. [^11^C](R)-PK11195 Autoradiography

**Figure 6:**
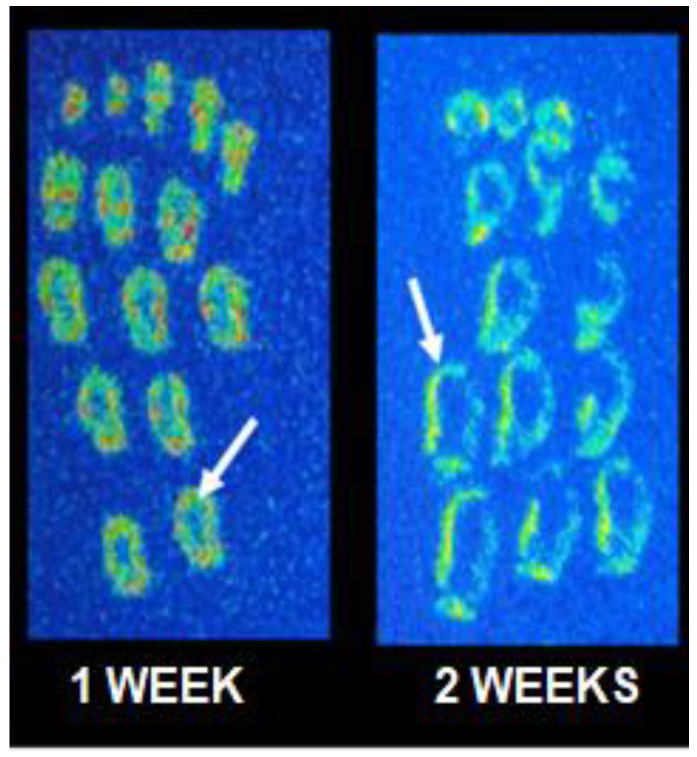
Illustrative [^11^C](*R*)-PK11195 autoradiography images of mammary murine tumor at 1 and 2 weeks after 4T1 cell inoculation. The white arrows indicate tumor edge uptake.

Visual evaluation of tumor sections showed that one week after inoculation of 4T1 cells, the [^11^C](*R*)-PK11195 uptake was present in the entire tumor volume, although with greater intensity at the tumor edge (Figure 6) and at 2 weeks, the uptake at the tumor edge was even more expressive.

### 3.4. Immunofluorescence

Tissue sections of murine mammary tumors obtained 1 and 2 weeks after inoculation showed the presence of the cell nucleus (Hoechst) throughout the tissue in all assays.

**Figure 7:**
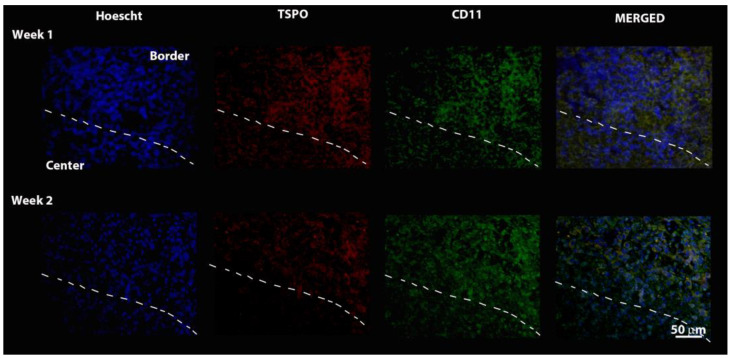
Images of immunofluorescence reactions in tumor tissue with Hoechst dye (Nucleus), TSPO, CD11 (macrophages), and fusion of all three. The dashed line divides the tissue sections into an upper (edge) and lower (center) region.

Immunofluorescence for CD11 macrophage and TSPO markers is shown in Figure 7. Macrophages (CD11) are distributed in all regions of the tumor tissue section, with greater expression at the tumor edge. The TSPO expression followed the same pattern, being more expressed at the border of the tumor at both time points, demonstrating an apparent decrease from 1 to 2 weeks.

**Figure 8:**
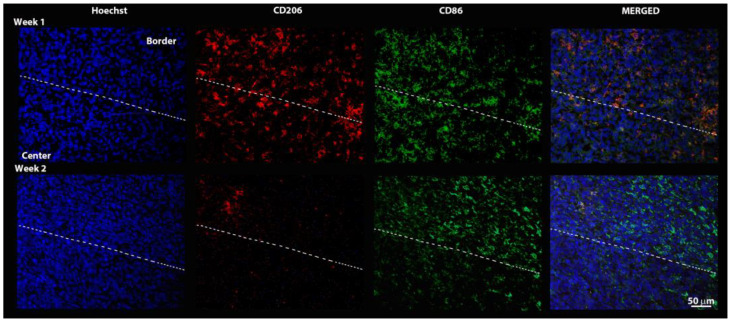
Immunofluorescence images representing tumor staining for Hoechst uptake (Nucleus), CD206 (macrophage M2, anti-inflammatory), CD86 (macrophage M1, pro-inflammatory), and fusion of all three. The dashed line divides the tissue section, where the upper region corresponds to the tumor edge and the lower region corresponds to the tumor center.

The immunofluorescence for pro- and anti-inflammatory macrophage phenotypes is shown in Figure 8. The anti-inflammatory phenotype (CD206) was slightly predominant in edge regions at 1 week, with an overall decrease in expression and accentuation of tumor edge predominance at 2 weeks. The pro-inflammatory phenotype (CD86) was expressed throughout the tumor in week 1, with a more evident higher concentration in the tumor edge region after 2 weeks.

**Figure 9:**
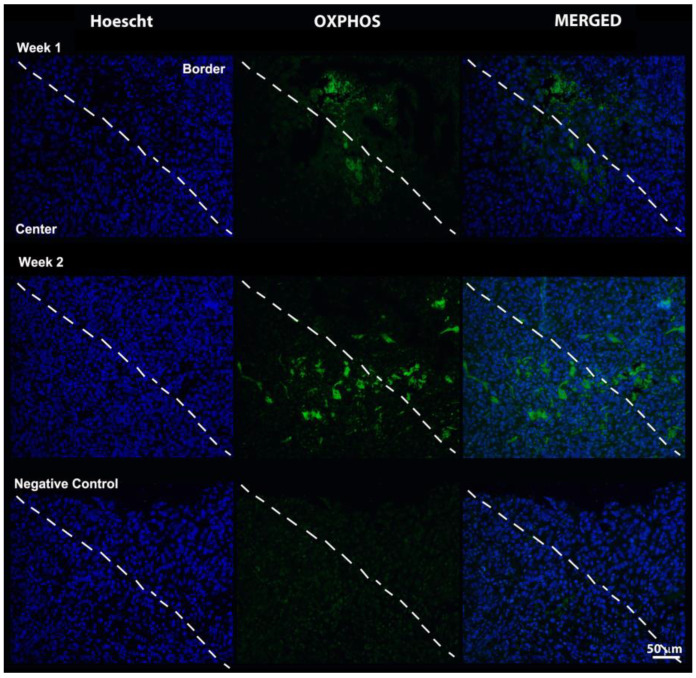
Images of immunofluorescence reactions in tumor tissue with Hoechst dye (Nucleus) and OXPHOS (oxidative phosphorylation), and fusion of the two of them. The dashed line divides the tissue sections into an upper (edge) and lower (center) region.

Figure 9 shows immunofluorescence with the mitochondrial oxidative phosphorylation marker antibody (OXPHOS). The OXPHOS had a decreased expression at 1 week relative to 2 weeks. At both time points, cell markings with OXPHOS were concentrated in the edge-center transition region.

### 3.5. Western Blot

**Figure 10:**
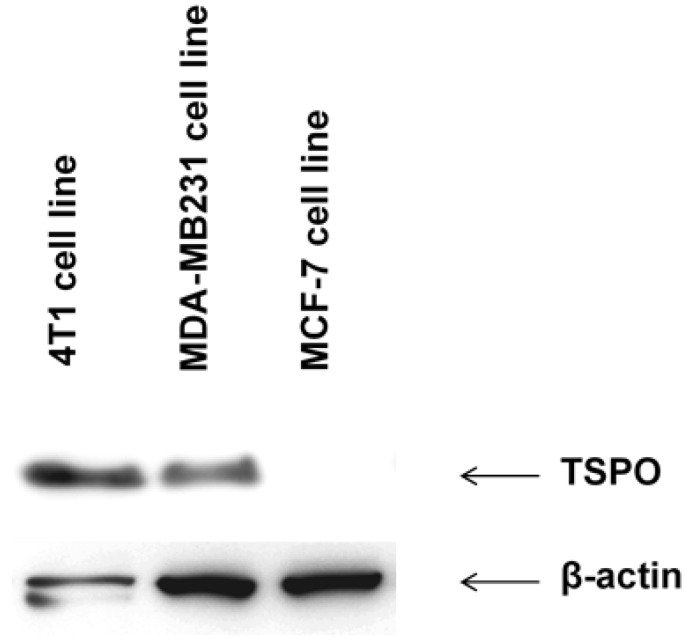
Western blot of TSPO expression in three cell lines of breast cancer (4T1, MDA-MB231, and MCF7).

Western Blot confirmed the expression of TSPO in the 4T1 cells, compared to other human breast cancer cell lines (Figure 10).

## 4. Discussion

The uptake of [^11^C](*R*)-PK11195 and [^18^F]FDG was evaluated in mammary tumors of female Balb/c mice 3 days, 1 and 2 weeks after inoculation of the highly tumorigenic and invasive 4T1 lineage [26]. Differences between [^11^C](*R*)-PK11195 and [^18^F]FDG uptake mechanisms suggest that visualization of the first tracer on a PET image was mainly related to the migration of inflammatory cells to the tumor microenvironment, while the uptake of [^18^F]FDG may be related to both the inflammation and tumor infiltrate.

For [^11^C](*R*)-PK11195, uptake decreased at 2 weeks compared to 1 week, while for [^18^F]FDG, uptake remained constant between the first and second weeks. From these results, we suggest that cellular glycolytic metabolism remains active while TME inflammatory cells occur in the first week and decrease over time, concomitantly with the serial increase in tumor cell metabolism, which would be primarily responsible for the uptake of [^18^F]FDG at 2 weeks. The [^11^C](*R*)-PK11195 TMR at three days suggests that initial inflammation is the predominant factor leading to uptake at this time point during the cell migration of the TAM.

The data from the control group, which represents the tracer uptake in a site injected only with medium (with no tumor cells), showed slightly higher uptake at 3 days that could be related to the mechanical injection injury, which was similar in the tumor group for both tracers. However, for 1 and 2 weeks, the tracer uptake was reduced compared to the 3 days-time point in the control group, although no significant differences were detected. The results from the site of medium injection show that the tracer behavior is different in the presence of tumor cells, reinforcing our hypothesis of tumor inflammation uptake specificity.

The PET images show that the uptake of both radiopharmaceuticals was more intense at the tumor edge. The [^11^C](*R*)-PK11195 autoradiography confirmed higher uptake in this region, which was more accentuated at 2 weeks, with almost undetectable central uptake in some tumor sections.

Immunofluorescence images reinforce the presence of TAMs in the breast cancer model used in our study, especially the pro-inflammatory type. This observation can be related to a study published by Viana et al. [27], where mice inoculated with 4T1 cells submitted to invasive procedures to create acute and chronic inflammatory environments, showed greater tumor progression in the presence of chronic inflammation than in acute inflammation. Furthermore, in the same study, the authors suggest that chronic cellular infiltration is important for tumor progression, as significantly higher populations of macrophages, dendritic cells, and lymphocytes were observed in mice with chronic inflammation.

Immunofluorescence images allowed us to observe macrophage cells within 1 week after inoculation of the tumor, with an apparently homogeneous distribution, regardless of phenotype. In the second week, there was a predominance of M1 (CD86+) pro-inflammatory cells, while M2 (CD206+) was diminished in relation to what was observed one week after the inoculation of 4T1 cells. Madera et al. [26] demonstrated in vitro that exposure to factors secreted by cells of the 4T1 lineage leads to increased macrophage responses after exposure to LPS (inflammatory response promoter), resulting in increased production of pro-inflammatory cytokines, phagocytosis, and release of nitric oxide. Classically activated macrophages (M1) are involved in Th1 responses, responsible for phagocytosing invading microorganisms and tumor cells, with the capacity to produce large amounts of pro-inflammatory cytokines [28], which may justify the expression of CD86 in both evaluated tumor sizes.

The anti-inflammatory M2 phenotype (CD206) was predominant in the tumor edge regions, with a decrease in expression from 1 week to 2 weeks, corroborating the finding by Yuancheng et al. [29], who identified greater CD206 immunofluorescence expression in M2 macrophages compared to M1 macrophages in an M1/M2 induced model in 4T1 cells. This same study demonstrated increased expression of cell labeling, with anti-CD206 in the tumor edge region compared to the tumor center. These findings agree with the recent study by Xiaoying Li et al. [30] who observed that TSPO expression occurs preferentially in activated (pro-inflammatory) macrophages, as well as decreased expression of CD206 in relation to CD86, which may imply the potentiality of TSPO-PET imaging as a technique for in vivo evaluation of infiltrating macrophages in the TME.

The temporal and spatial variations in M1, M2, and TSPO expression using immunofluorescence markers are consistent with the tumor distribution of [^11^C](*R*)-PK11195 observed on our PET imaging and autoradiography.

The TSPO is expressed in macrophages, however, 4T1 cells may also express TSPO, as confirmed by Western blot in this study. Expression of TSPO is well recognized in different solid tumors, including breast cancer [31], and may be involved in the disruption in morphology and migration of mammary cancer cells [32]. Hence, there could be an overlap of inflammatory and tumor uptake mechanisms not only for [^18^F]FDG but also for [^11^C](*R*)-PK11195. The significant reduction in TSPO radioligand uptake in the 4T1 mammary cancer model after macrophage depletion suggests that the inflammatory uptake is predominant in this tumor [33]. The predominance of macrophage uptake of [^11^C](*R*)-PK11195 on PET is reinforced in this study by the congruence in the cell expression of TSPO and CD11 markers.

The evaluation with anti-OXPHOS aimed to understand the oxidative phosphorylation in the mitochondria of several cells of the organism, including tumor cells [34], therefore, the use of the immunofluorescence technique associated with the use of anti-OXPHOS was a way to verify the viability of 4T1 tumor cells in the time point evaluated. The results demonstrated the uptake of anti-OXPHOS in the regions of low expression of CD206 and TSPO in two weeks, suggesting that there is no false-negative labeling of mitochondria by the TSPO antibody, which corresponds to the presence of the translocator protein of 18 kDa in tumor-associated macrophages, and makes it possible to maintain the interpretation that pro-inflammatory cells are the cells with greatest infiltration in tumors of larger volumes.

The limitations of this study include that only the minority of animals were evaluated by the two radiotracers at the three time-points, impeding a repeated measures analysis an also the impossibility of quantifying cell staining in tumor tissues due to technical issues. The absence of these data made it impossible to quantitatively corroborate the findings with PET results. There was also heterogeneity in tumor volumes at 1 and 2 weeks, related to the individual growth of each tumor, which could interfere with the TAM based on the tumor size.

The [^11^C](*R*)-PK11195 PET is primarily used for the detection of activated microglia in neuroinflammation, with secondary use for the investigation of other inflammatory conditions. In this context, the present work brings a new and important contribution to understanding of the dynamics of tracer uptake by tumor-associated macrophages and the relationship with tumor development in an experimental model of mammary cancer.

## 5. Conclusions

The use of [^11^C](*R*)-PK11195 as a marker of tumor inflammation identified heterogeneous tumor inflammation in a murine model of breast cancer. Immunofluorescence confirms that both pro-inflammatory and anti-inflammatory macrophages were observed mainly in the tumor edge, which is the same region that expresses TSPO and shows greater [^11^C](*R*)-PK11195 uptake on PET imaging and autoradiography. These results suggest that the interaction of TAM in the tumor microenvironment is heterogeneous and varies according to tumor size. Together with the glycolytic marker, [^18^F]FDG, molecular imaging with [^11^C](*R*)-PK11195 may provide better characterization of metabolic and inflammatory responses in cancer.

## Data Availability

Data from this study is available from the corresponding author upon reasonable request.

## Supplementary Materials

The following supporting information can be downloaded at: https://www.mdpi.com/article/10.3390/pharmaceutics14122715/s1. Additional information about animals diet is provided.
