# Supplementary material for: Potential of [11C](R)-PK11195 PET Imaging for Evaluating Tumor Inflammation: A Murine Mammary Tumor Model
Source: Pharmaceutics. 2022 Dec 4;14(12):2715. doi: 10.3390/pharmaceutics14122715 (PMC9786563; doi:10.3390/pharmaceutics14122715)
Supplement: Supplementary file 1 [file pharmaceutics-14-02715-s001.zip › pharmaceutics-2020142-supplementary.pdf]

Supplementary Materials: Potential of [^{11}C](*R*)-PK11195 PET Imaging for Evaluating Tumor Inflammation: A Murine Mammary Tumor Model

Aline Morais de Souza [1], Caroline Cristiano Real [1,2], Mara de Souza Junqueira [3], Larissa Estessi de Souza [1], Fábio Luiz Navarro Marques [1], Carlos Alberto Buchpiguel [1], Roger Chammas [3], Marcelo Tatit Sapienza [1] and Daniele de Paula Faria [1,*]

[1] Laboratory of Nuclear Medicine (LIM 43), Department of Radiology and Oncology, Faculdade de Medicina FMUSP, Universidade de Sao Paulo, Sao Paulo 05403-911, Brazil

[2] Department of Nuclear Medicine and PET Center, Aarhus University Hospital, DK-8200 Aarhus, Denmark

[3] Centro de Investigação Translacional em Oncologia (CTO), Instituto do Câncer de Sao Paulo (ICESP), Hospital das Clínicas HCFMUSP, Faculdade de Medicina, Universidade de Sao Paulo, Sao Paulo 01246-000, Brazil

* Correspondence: danielefaria1@gmail.com

Diet details:

Vegetable fat source: degummed soybean oil. Guarantee levels per kilogram of product: MOISTURE (max) 125 g/kg; MINERAL MATERIAL (max) 90 g/kg; CALCIUM (min-max) 10-14 g/kg; CRUDE PROTEIN (min) 220 g/kg; FIBROUS MATTER (max) 70 g/kg; PHOSPHORUS (min) 8,000mg/kg; ETHERE EXTRACT (min) 40 g/kg; GROSS ENERGY (MIN) 3,800 kcal/kg. VITAMINS: VITAMIN A (min) 25,500 IU/kg; VITAMIN D3 (min) 2,100 IU/kg; VITAMIN E (min) 60 IU/kg; VITAMIN K3 (min) 12.5 mg/kg; VITAMIN B1 (min) 14.4 mg/kg; VITAMIN B2 (min) 11 mg/kg; VITAMIN B6 (min) 12 mg/kg; VITAMIN B12 (min) 60 mcg/kg; NIACIN (min) 60 mg/kg; PANTOTHENIC ACID (min) 112 mg/kg; FOLIC ACID (min) 6 mg/kg; BIOTIN (min) 0.2 mg/kg; CHOLINE (min) 2,400 mg/kg. MINERALS: SODIUM (min) 2,700 mg/kg; IRON (min) 50 mg/kg; MANGANES (min) 60 mg/kg; ZINC (min) 60 mg/kg; COPPER (min) 10 mg/kg; IODINE (min) 2 mg/kg; SELENIUM (min) 0.05 mg/kg; COBALT (min) 1.5 mg/kg; FLUORINE (max) 80 mg/kg. AMINO ACIDS: LYSINE (min) 14 g/kg; METHIONINE (min) 5,000 mg/kg. ADDITIVES: BHT 100 mg/kg.
